# Characteristics of Plasmonic Bragg Reflectors with Graphene-Based Silicon Grating

**DOI:** 10.1186/s11671-016-1633-0

**Published:** 2016-09-22

**Authors:** Ci Song, Xiushan Xia, Zheng-Da Hu, Youjian Liang, Jicheng Wang

**Affiliations:** 1 School of Science, Jiangsu Provincial Research Center of Light Industrial Optoelectronic Engineering and Technology, Jiangnan University, Wuxi, 214122 China; 2 Key Laboratory of Semiconductor Materials Science, Institute of Semiconductors, Chinese Academy of Sciences, 912, Beijing, 100083 China

**Keywords:** Plasmonics, Bragg reflectors, Graphene-based, FEM

## Abstract

We propose a plasmonic Bragg reflector (PBR) composed of a single-layer graphene-based silicon grating and numerically study its performance. An external voltage gating has been applied to graphene to tune its optical conductivity. It is demonstrated that SPP modes on graphene exhibit a stopband around the Bragg wavelengths. By introducing a nano-cavity into the PBR, a defect resonance mode is formed inside the stopband. We further design multi-defect PBR to adjust the characteristics of transmission spectrum. In addition, through patterning the PBR unit into multi-step structure, we lower the insertion loss and suppress the rippling in transmission spectra. The finite element method (FEM) has been utilized to perform the simulation work.

## Background

Surface plasmon polaritons (SPPs) are surface waves that propagate along the boundary surface between dielectric and metallic materials with fields decaying exponentially in both sides, thereby creating the subwavelength confinement of electromagnetic waves [1]. These are mainly electromagnetic modes resulting from the resonant interaction between light waves and the collective electron oscillations, which leads to its unique properties [2]. Plasmonic nanostructures offer the potential to overcome diffraction limits in dielectric structures, enabling us to miniaturize optical devices [3]. For example, plasmonic has been widely researched in integrated photonic circuits [4], photonic crystals [5], optical antennas [6, 7], nano-laser [8], data recording [9], filters [10], refractive index sensor [11], biological sensors [12], metalens [13], plasmonic lens [14], and so forth. Among the structures based on SPPs, the metal-insulator-metal (MIM) structure has been investigated extensively in designing plasmonic Bragg reflector. For example, periodic changes in the dielectric materials of the MIM waveguides have been proposed to design effective filtering around the Bragg frequency [15]; the thick-modulated and index-modulated Bragg reflectors have been reported to widen bandgap [16]; metal-embedded MIM structure also has been studied to improve the performance of conventional step profile MIM plasmonic Bragg reflectors (PBRs) [17]. However, plasmonic materials, usually noble metals, are hardly tunable and have great ohmic losses at the wavelength regimes of interest, therefore limiting their potential for some specific applications.

Graphene, a single layer of carbon atoms densely arranged into a honeycomb pattern, has been widely explored as a newly alternative to plasmonic material [18, 19]. Graphene plasmonics, similar to metal plasmonics at the visible region, can be easily induced in the near-infrared to terahertz (THz) regime. In particular, the surface charge density, namely chemical potential, can be actively modified by chemical doping or external gate voltage, thus giving rise to dramatic changes in the optical properties [20]. Additionally, SPPs bound to graphene display a strong field confinement, already verified by experiments [21, 22]. These remarkable and outstanding properties in turn enable a utility optical material in optoelectronic applications. In recent years, great attention has been focused on graphene-based plasmonic waveguides [23–27]. de Abajo et al. have researched the propagation properties of graphene plasmonic waveguide constituted by individual and paired nanoribbons [28]. The tunable nano-modulators based on graphene plasmonic waveguide modulators have been proposed and numerically demonstrated [29]. Lu et al. have designed a slow-light waveguide based on graphene and silicon-graded grating [30]. Wang et al. have utilized a graphene waveguide achieving a tunable plasmonic Bragg reflector [31].

In this paper, we propose a PBR structure consisting of a single-layer graphene and silicon grating and numerically study its performance. We employ a silica spacer layer to separate the monolayer graphene and silicon grating and an external voltage gating to tune the surface conductivity of graphene. The finite element method (FEM) [32] has been utilized to perform the simulation work. We demonstrate that SPP modes on graphene exhibit a stopband around the Bragg wavelengths. Based on Bragg scattering condition, several modulation schemes have been used to adjust the characteristics of transmission spectrum. Furthermore, we introduce a defect into PBR, consequently realizing a resonant defect mode with a high and tunable Q factor. Based on the discussion of one defect cavity, we further study the multi-defect cases. At last, the PBR unit is designed into a multi-step pattern to reduce the rippling sidelobes and insertion loss. Such proposed designs, we believe, may help build some actively tunable modulators.

## Methods

**Fig. 1:**
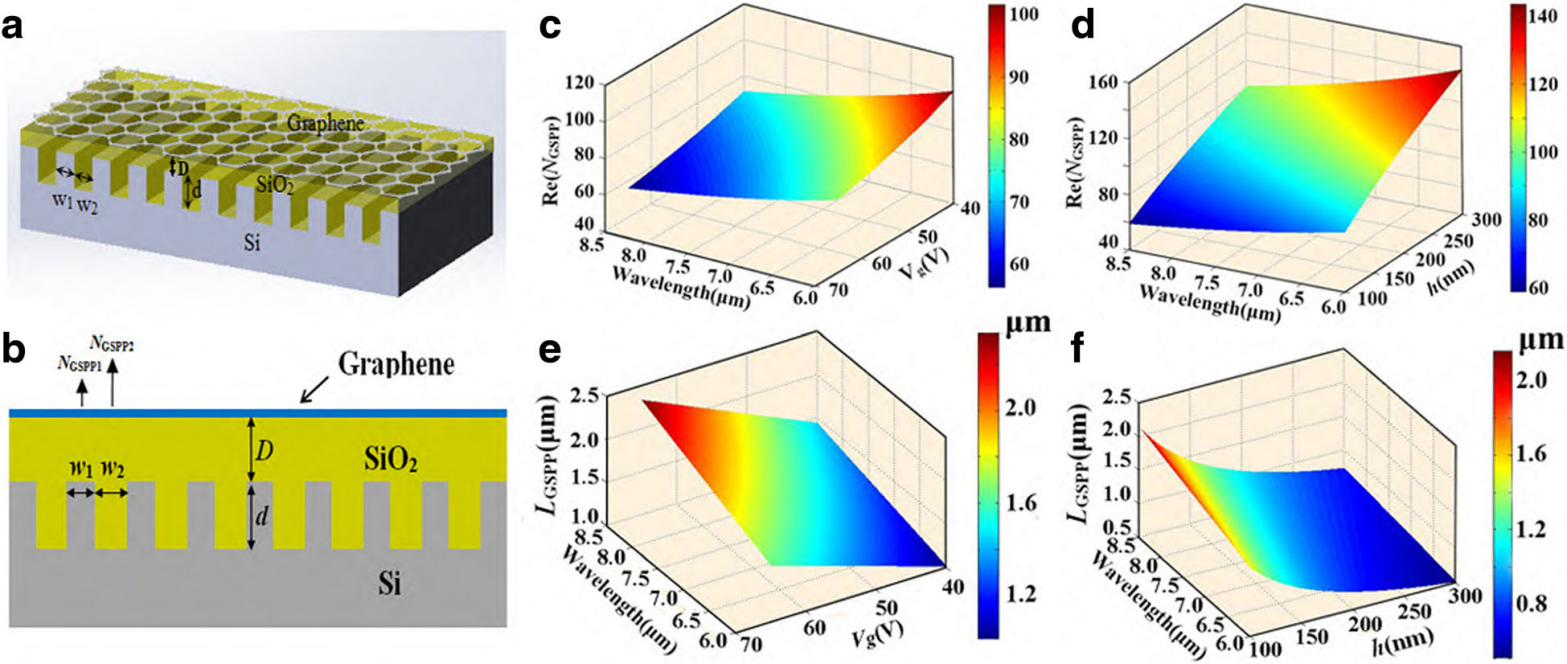
**a**, **b** 3D and 2D schematic illustration of PBR. A single layer of graphene and a silicon (Si) grating substrate between which a silica (*SiO*_*2*_) layer has been embedded. The Si substrate has a groove grating structure with period number *N*, period *w*_1_+ *w*_2_, groove width *w*_2_, and groove depth *d*. The constant distance (*D*) between graphene and Si grating is 100 nm. **c**, **d** The real part of effective refractive index (*Re*(*N*_eff_)) in relation to the gate voltage (*V*_*g*_) and the thickness of silica layer (*h*). **e**, **f** The propagation distance (*L*_GSPP_) in relation to the gate voltage (*V*_*g*_) and the thickness of silica layer (*h*)

As plotted in Fig. 1a, the proposed PBR in this work is composed of a single-layer graphene and a silicon grating substrate between which a silica layer has been embedded. And major structural parameters are labeled in Fig. 1a, b. In our investigation, the incident light at mid-infrared regime intrigues an excitation of a transverse magnetic (TM)-polarized SPP mode propagating along the graphene sheet. By solving the Maxwells equations with boundary conditions [33], we obtain the dispersion relation for TM modes supported on the graphene layer which is surrounded with air and silica:

1 $$ \frac{\varepsilon_{\mathrm{Air}}}{\sqrt{{k_{\mathrm{GSPP}}}^2-\frac{\varepsilon_{\mathrm{Air}}{\omega}^2}{c^2}}}+\frac{\varepsilon_{{\mathrm{SiO}}_2}}{\sqrt{{k_{\mathrm{GSPP}}}^2-\frac{\varepsilon_{{\mathrm{SiO}}_2}{\omega}^2}{c^2}}} = -\frac{\sigma \left(\omega,\ {k}_{\mathrm{GSPP}}\right)i}{\omega {\varepsilon}_0} $$

Since we only consider the non-retarded regime (*k*_GSPP_ >> ω), the Eq. (1) can be simplified to

2 $$ {k}_{\mathrm{GSPP}} \approx {\varepsilon}_0\frac{\varepsilon_{\mathrm{Air}}+{\varepsilon}_{{\mathrm{SiO}}_2}}{2}\frac{2i\omega }{\sigma \left(\omega, {k}_{\mathrm{GSPP}}\right)} $$

Here, *k*_GSPP_ is the wave vector of SPPs on graphene layer, and the dielectric constants of air and SiO_2_ are assumed to be 1 and 3.9, respectively. The optical conductivity of graphene is *σ*(*ω*, *k*_GSPP_) determined by Kobo formula [34]. At the mid-infrared frequency range, *σ*(*ω*, *k*_*GSPP*_) can be simplified into a Drude-like equation [35]:

3 $$ \sigma \left(\omega \right) = \frac{e^2{\mu}_c}{\pi {\hslash}^2}\frac{i}{\omega +i{\tau}^{-1}} $$

In graphene layer, *τ* denoting the relaxation time can be expressed as *μμ*_*c*_/(*ev*_*f*_^2^), which relates to the carrier mobility *μ* and Fermi velocity *v*_*f*_ = 10^6^ m/s [36]. The carrier mobility is reasonably chosen to be *μ =* 20,000 cm^2^ V^−1^ s^−1^ from experiment results [37]. And, *μ*_*c*_ = *ħv*_*f*_(*πn*)^1/2^ is the chemical potential where the surface charge carrier density is expressed as *n* = *ε*_*0*_*ε*_*d*_*V*_*g*_/(*eh*) [18]. Here, *ε*_0_ and *ε*_*d*_ are the dielectric constants of free space and SiO_2_, respectively. *V*_*g*_ is the applied gate voltage, *e* is the electron charge, and *h* is the thickness of silica layer. In Fig. 1a, b, *h* equals to *D* at non-groove sections and *h* equals to *D* + *d* at groove sections. This expression also indicates that the chemical potential can be induced by not only a voltage gate but also the thickness of silica layer. From the above equations, a more specific graphene plasmonic dispersion relation is obtained as follows:

4 $$ {k}_{\mathrm{GSPP}} = \frac{\pi \hslash {\varepsilon}_0\left({\varepsilon}_{\mathrm{Air}}+{\varepsilon}_{{\mathrm{SiO}}_2}\right)}{e^2{v}_f{\left(\pi \frac{\varepsilon_0{\varepsilon}_{Si{O}_2}{V}_g}{eh}\right)}^{\frac{1}{2}}}\left(1+\frac{i}{\tau \omega}\right){\omega}^2 $$

Another important parameter derived from the above equation is *N*_GSPP_ = *k*_GSPP_/*k*_0_—the effective refractive index of GSPP, which shows the ability to confine GSPP on graphene. The propagation length is defined as *L*_GSPP_ = 1/[2*k*_0_Im(*N*_GSPP_)] featuring the GSPP propagation loss. Throughout the paper, the influence of substrate silicon on the dispersion relation is negligible when the silica layer is above 100 nm [30]. The dependence of *Re*(*N*_GSPP_) and *L*_GSPP_ on the gate voltage *V*_*g*_ and the thickness of silica layer *h* are illustrated in Fig. 1c–f where the wavelength range is 6 to 9 μm. Obviously, from Fig. 1c, d, the *Re*(*N*_GSPP_) shows a significant increase when the gate voltage *V*_*g*_ decreases or the thickness of silica layer *h* goes up. In Fig. 1e, f, however, a longer propagation distance is achieved by a growing *V*_*g*_ or a decreasing *h*. Hence, the two important factors should be both taken into consideration for our designs.

Generally, for wavelength-sensitive operations, a plasmonic Bragg reflector is constructed by periodically modulating the effective refractive index of the waveguide. There are some popular accesses to achieve this such as width modulation [38] and refractive index modulation [39]. In our work, a graphene-based Bragg reflector is formed by periodically modulating *N*_GSPP_. According to the aforesaid discussion, this can be realized by alternatively varying the thickness of silica layer yielding a silicon grating substrate, shown in Fig. 1a, b. Thus, the Bragg scattering condition [39] in our case can be formulated as:

5 $$ {w}_1\mathrm{R}\mathrm{e}\left({N}_{\mathrm{GSPP}1}\right)+{w}_2\mathrm{R}\mathrm{e}\left({N}_{\mathrm{GSPP}2}\right)=m\frac{\lambda_b}{2} $$

Here, *λ*_*b*_ is the Bragg wavelength and *m* is an integer assumed to be 1 in our discussion. *N*_GSPP1_ and *N*_GSPP2_ are the effective refractive index of GSPP on differently doped areas of graphene, respectively (see Fig. 1a, b). The Bragg wavelength will be stopped when Eq. (5) is satisfied.

## Results and discussion

**Fig. 2:**
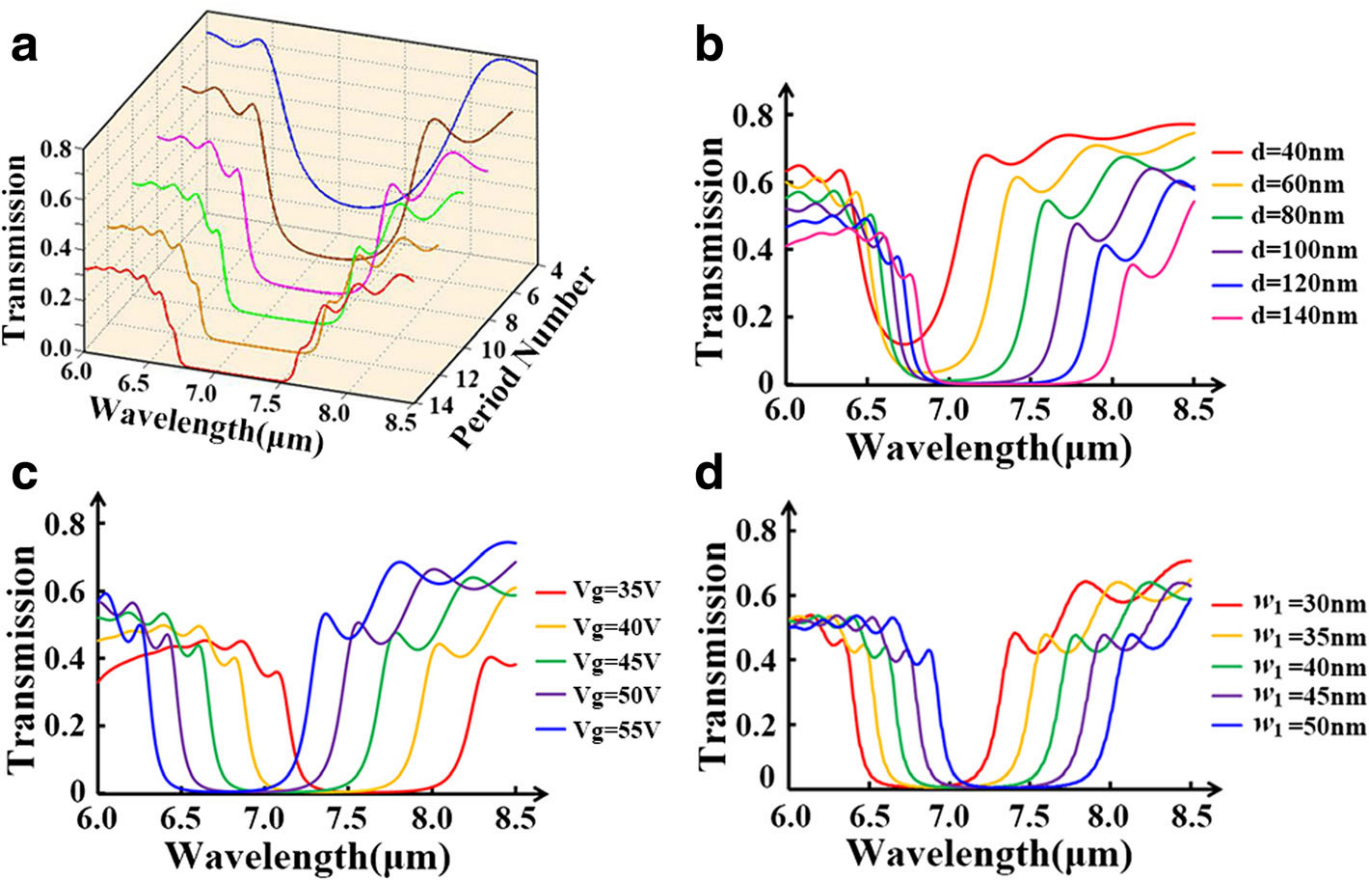
Simulated transmission spectra. **a** The PBR with the different period numbers for the groove depth *d* = 100 nm, the grating period *w*_1_ + *w*_2_ = 80 nm (*w*_1_ = *w*_2_), and the gate voltage *V*_*g*_ = 45 V. **b** The PBR with the different groove depth for the period number *N* = 8, the grating period *w*_1_ + *w*_2_ = 80 nm (*w*_1_ = *w*_2_), and the gate voltage *V*_*g*_ = 45 V. **c** The PBR with the different gate voltages for the period number *N* = 8, the groove depth *d* = 100 nm, and the grating period *w*_1_ + *w*_2_ = 80 nm (*w*_1_ = *w*_2_). **d** The PBR with the different *w*_1_ for the period number *N* = 8, the groove depth *d* = 100 nm, and the gate voltage *V*_*g*_ = 45 V

At first, we discuss the influence of period number on transmission spectra of PBR. The parameters are set as *w*_1_ = *w*_2_ = 40 nm, *D* = *d* = 100 nm, and *V*_g_ = 45 V. The period number *N* is ranged from 4 to 14. The simulated transmission spectra for different period numbers are shown in Fig. 2a. As the period number decreases, the propagation loss is lower whereas the stopband is narrower. To balance these two trends, we choose period number to be 8 in the following discussion. Besides, these spectra all display some sidelobes outside the stopband caused by light scattering at the end of PBR. Next, we study the effects of grating groove depth *d* and gate voltage *V*_g_ on the operating wavelengths of PBR. Figure 2b indicates a pronounced red-shift of Bragg wavelengths and a widened stopband with a growing groove depth *d*; nevertheless, the transmission is gradually lowered as the groove depth *d* increases. In Fig. 2c, we see a blue-shift of Bragg wavelengths and a growing transmission when the gate voltage goes up. The shifting effect can be attributed to the alteration of *N*_GSPP_ with an increasing groove depth *d* or a growing gate voltage *V*_g_. And the modification of *L*_GSPP_ by varying groove depth *d* or gate voltage *V*_g_ accounts for the change of transmission above. We further study another two parameters from the Bragg condition, *w*_1_ and *w*_2_. As displayed in Fig. 2d, we see a noticeable red-shift of the central wavelengths while the width of stopband is almost the same when *w*_1_ is increasing. By varying *w*_2_, the transmission spectra show almost similar features.

**Fig. 3:**
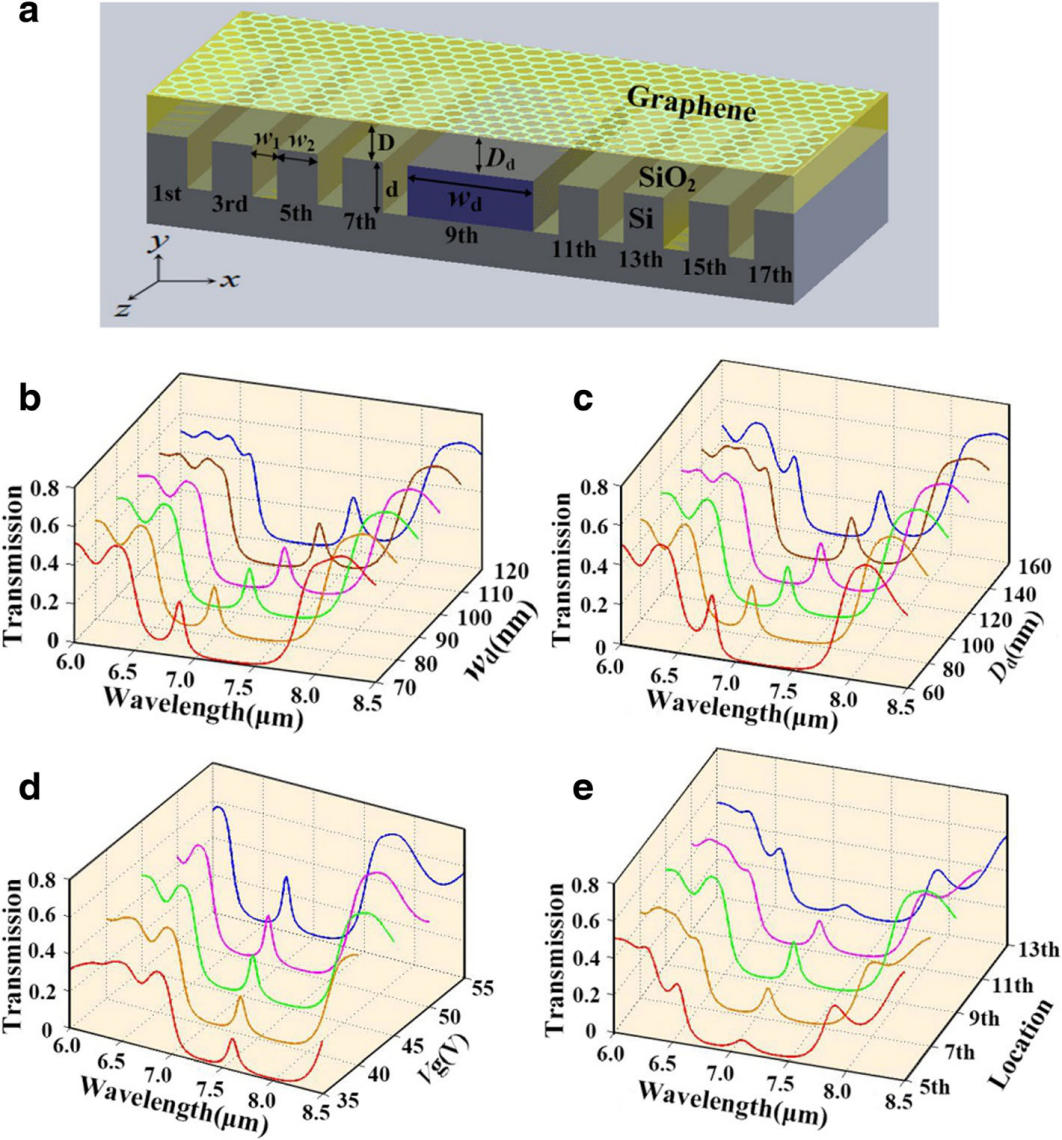
**a** 3D schematic illustration of PBR by introducing a defect cavity located at ninth site with the width *w*_*d*_ and the distance *D*_*d*_ between graphene and Si. **b** Transmission spectra of the defecting PBR with different *w*_*d*_, *D*_d_ = 100 nm, and the gate voltage *V*_*g*_ = 45 V. **c** Transmission spectra of the defecting PBR with different *D*_*d*_, *w*_*d*_ = 90 nm, and the gate voltage *V*_*g*_ = 45 V. **d** Transmission spectra of the defecting PBR with different *V*_*g*_, *w*_*d*_ = 90 nm, and *D*_*d*_ = 100 nm. **e** Transmission spectra of the defecting PBR with different defect locations, *w*_*d*_ = 90 nm, *D*_*d*_ = 100 nm, and *V*_*g*_ = 45 V

In addition, we introduce a nano-cavity into the plasmonic Bragg grating causing a defect in the PBR’s periodicity. At some specific wavelengths, resonant modes will be formed around the nano-cavity, and accordingly, there will be a transmission peak inside the stopband [40]. In Fig. 3a, we set the nano-cavity in the center of PBR and as same as that in the study above. The thickness of silica layer below the nano-cavity graphene is set as *D*_*d*_ = 100 nm, and firstly, we discuss the length of defect cavity *w*_d_ ranging from 70 to 120 nm. In Fig. 3b, we see clear peaks at the stopping range of transmission spectra as we have expected. By lengthening the nano-cavity step by step, we find an obvious red-shifting of the resonant defect modes with the stopband unmoving. Secondly, we fix the length of nano-cavity as 90 nm and research the influence of *D*_*d*_ on the defect modes. The simulated results plotted in Fig. 3c present similar characteristics to that in Fig. 3b. Therefore, we have an access to tune the defect modes by varying the length of nano-cavity or the thickness of silica layer below the nano-cavity graphene. Next, we employ varied gating voltage on graphene sheet, and consequently, the spectra response is shifting as a whole, exhibited in Fig. 3d. Lastly, we set the defect cavity at different locations and study the features of transmission spectra. In Fig. 3e, defect cavity is moved from one side to the center. To get into more principles behind the off-to-on effect, we plot the electric field profiles at *λ* = 7.105 μm for the three cases (defect at third, fifth, and seventh location sites).

**Fig. 4:**
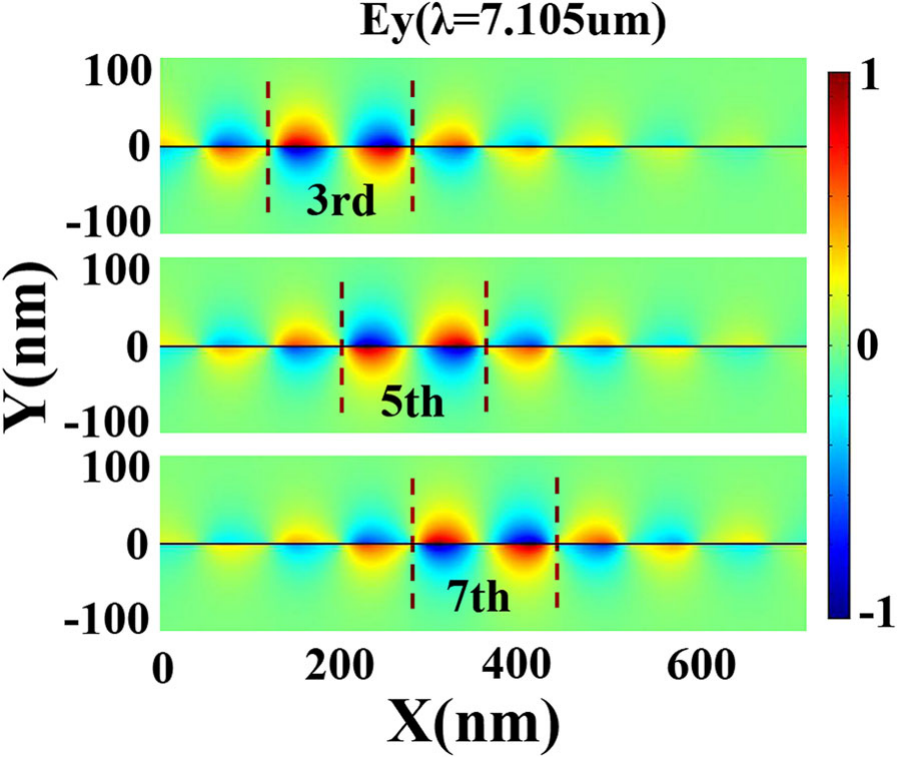
The electric field profiles (*E*_*y*_) for the defecting PBR with different defect locations, *λ* = 7.105 μm, *w*_*d*_ = 90 nm, *D*_*d*_ = 100 nm, and *V*_*g*_ = 45 V. The *dashed lines* denote the defect-mode areas

As shown in Fig. 4, pronounced resonant modes form at defect regions dividing the original Bragg reflector into two new Bragg reflectors. When the reflection by the two new Bragg reflectors adds up destructively, a high transmission occurs. However, when the two new Bragg reflectors are different from each other, the reflected beams from them usually cannot perform a well-destructive interference, which explain the off-to-on effect.

**Fig. 5:**
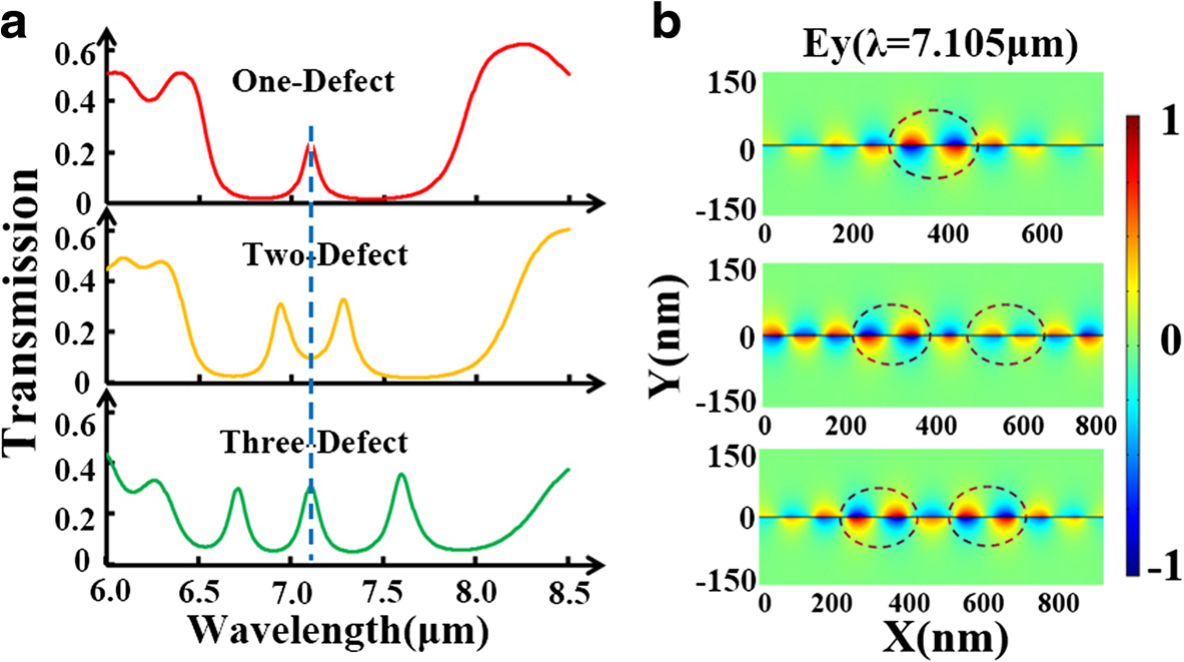
**a** Transmission spectra of the defecting PBR at three different cases: one-defect, two-defect, and three-defect. **b** The electric field profiles (*E*_*y*_) at *λ* = 7.105 μm for these three cases. The *red dashed circles* denote the resonant areas

**Fig. 6:**
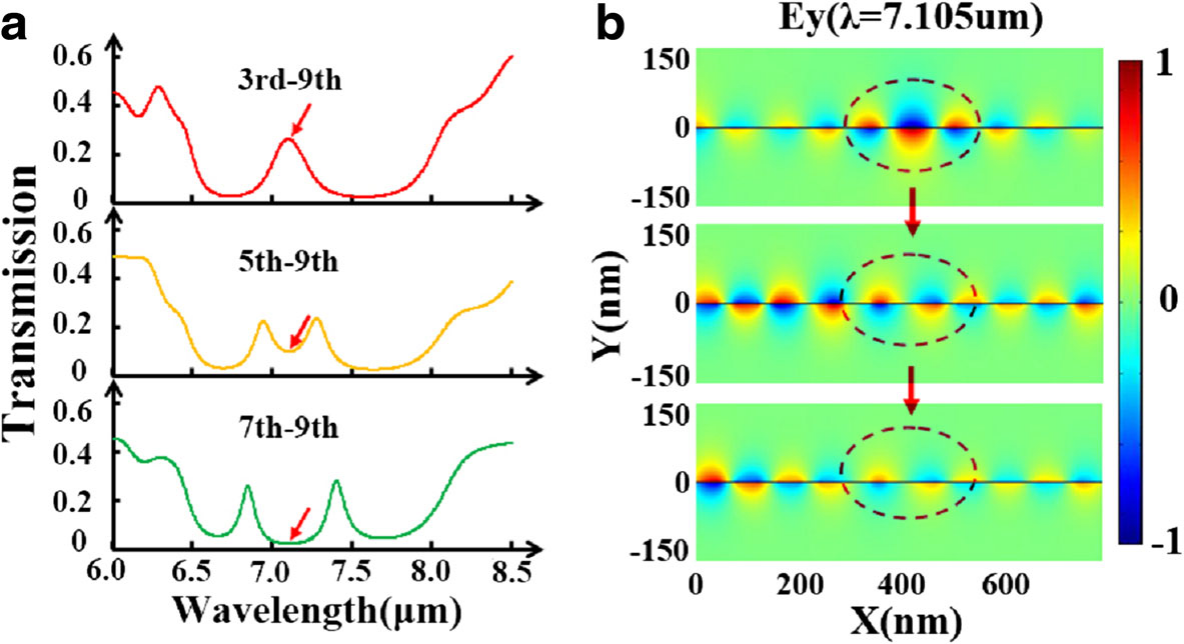
**a** Transmission spectra of the PBR with double defect for three different location combinations: third–ninth, fifth–ninth, and seventh–ninth. **b** The electric field profiles (*E*_*y*_) for these three cases at *λ* = 7.105 μm (pointed by the *arrows* in transmission spectra). The *red dashed circles* denote the central defect-mode region

**Fig. 7:**
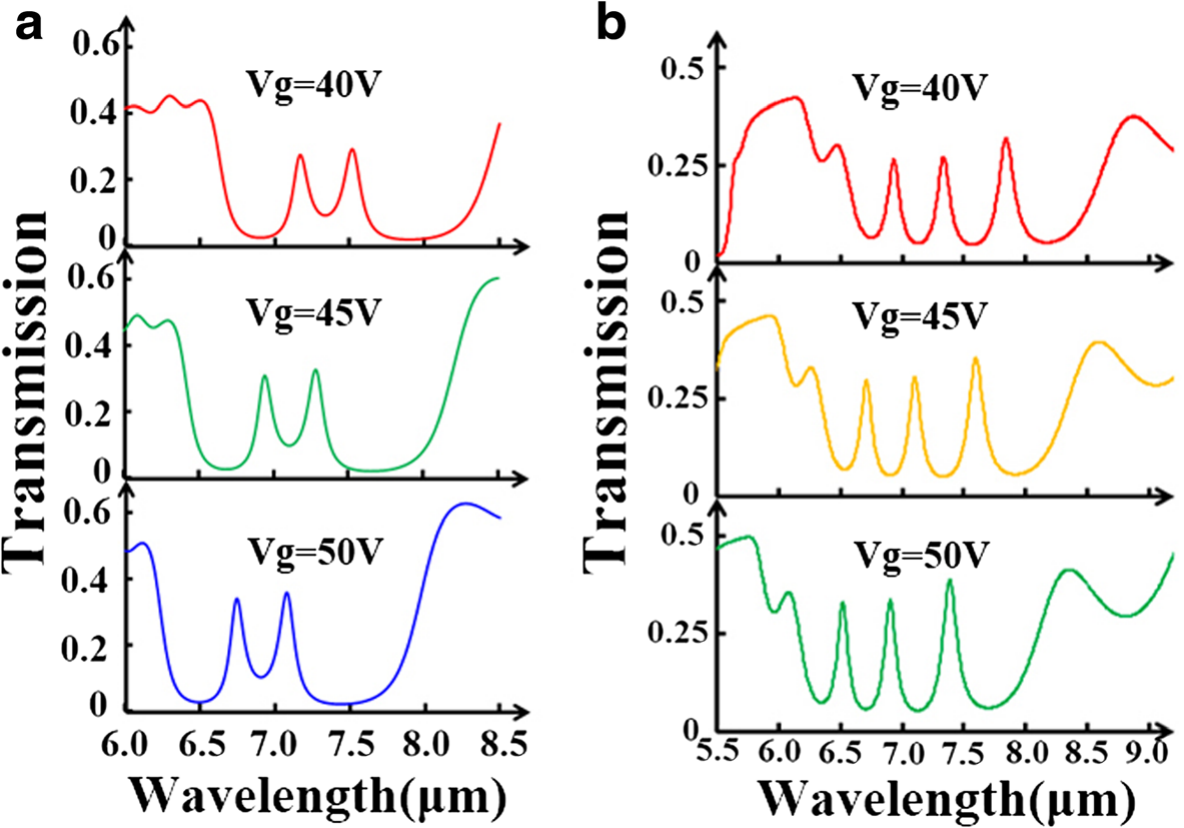
**a** Transmission spectra of the PBR with two defects for different gate voltages. **b** Transmission spectra of the PBR with three defects for different gate voltages

Since the defect cavity works differently when they are located at different sites, we further propose multiple defect cavities. At first, we compare the transmission spectra for three cases: one-defect, two-defect, and three-defect in PBR. All defect cavities are set at the same condition: *w*_*d*_ = 90 nm and *D*_*d*_ = 100 nm. Through Fig. 5a, it is demonstrated that multiple transmission peaks inside the stopband can be induced by adding the number of defect cavity in PBR. In Fig. 5b, we plot the electric field profiles at *λ* = 7.105 μm for these three cases. The red dashed circles denoting the resonant areas indicate that these defect modes from each nano-cavity will interfere with each other leading to these amazing features in transmission spectra. Besides, stopband can be greatly widened with multiple defect cavities. Then, we place one defect cavity in the center and place another one at different locations. Figure 6a points out that there is a notable splitting of defect-resonance mode in the two-defect case. It should be noted, however, that this splitting effect is only achieved when these two defect cavities are close enough to each other. As illustrated in Fig. 6b, the closer these two defects are together, the stronger the destructive interference between them, which gives rise to the splitting effect. Furthermore, we apply various gate voltages to the two-defect case producing a shifting effect of the multiple transmission peaks, seen in Fig. 7a. Similar shifting effect is also found in the three-defect case by varying gate voltage, shown in Fig. 7b.

**Fig. 8:**
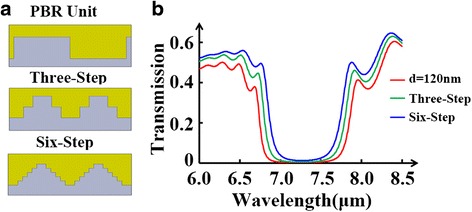
**a** 2D schematic illustration of PBR unit designed into a multi-step pattern: a three-step PBR unit and a six-step PBR unit, respectively. **b** Transmission spectra of PBR for three different cases. The *red line* represents the original PBR design (groove depth *d* = 120 nm); the *green* one and the *blue* one denote the three-step and six-step pattern cases, respectively

In the end, we design the PBR unit into a multi-step pattern to deal with the high insertion loss and severe rippling in transmission spectra resulting from the abrupt change of *N*_GSPP_ in the groove depth [38]. A three-step and a six-step version of PBR unit are well illustrated in Fig. 8a. After comparing the transmission spectra in Fig. 8b, we find the expected enhancement on transmission spectra and rippling suppression. Additionally, the stopband is gradually narrowed when the PBR unit is changed into more steps. The multiple steps in a PBR unit actually adding multiple reflections into the Bragg reflection process make it harder to satisfy the Bragg condition, which results in the sidelobe suppression and narrowed stopband.

## Conclusions

In conclusion, we design a PBR structure consisting of a single-layer graphene and silicon grating and numerically study its performance. We employ an external voltage gating to tune the surface conductivity of graphene. It is found that SPP modes on graphene exhibit a stopband around the Bragg wavelengths. Based on Bragg scattering condition, we discuss several modulation schemes to adjust the characteristics of transmission spectrum. Furthermore, we introduce a nano-cavity into PBR, consequently realizing a resonant defect mode. We further propose multi-defect PBR and achieve multiple peaks inside the stopband. At last, by designing the PBR unit into multi-step pattern, we lower the insertion loss and suppress the rippling in transmission spectra. We hope all the proposed designs above can help pave new ways in actively tunable modulation application.

## Abbreviations

FEM: Finite element method
PBR: Plasmonic Bragg reflector
SPPs: Surface plasmon polaritons
THz: Terahertz
TM: Transverse magnetic
